# Unsupervised Domain Adaptation for Classification of Histopathology Whole-Slide Images

**DOI:** 10.3389/fbioe.2019.00102

**Published:** 2019-05-15

**Authors:** Jian Ren, Ilker Hacihaliloglu, Eric A. Singer, David J. Foran, Xin Qi

**Affiliations:** ^1^Department of Electrical and Computer Engineering, Rutgers University, Piscataway, NJ, United States; ^2^Department of Biomedical Engineering, Rutgers University, Piscataway, NJ, United States; ^3^Section of Urologic Oncology, Rutgers Cancer Institute of New Jersey, New Brunswick, NJ, United States; ^4^Center for Biomedical Imaging and Informatics, Rutgers Cancer Institute of New Jersey, New Brunswick, NJ, United States

**Keywords:** histpathology, unsupervised domain adaptation, color normalization, adversarial training, convolutional neural networks

## Abstract

Computational image analysis is one means for evaluating digitized histopathology specimens that can increase the reproducibility and reliability with which cancer diagnoses are rendered while simultaneously providing insight as to the underlying mechanisms of disease onset and progression. A major challenge that is confronted when analyzing samples that have been prepared at disparate laboratories and institutions is that the algorithms used to assess the digitized specimens often exhibit heterogeneous staining characteristics because of slight differences in incubation times and the protocols used to prepare the samples. Unfortunately, such variations can render a prediction model learned from one batch of specimens ineffective for characterizing an ensemble originating from another site. In this work, we propose to adopt unsupervised domain adaptation to effectively transfer the discriminative knowledge obtained from any given source domain to the target domain without requiring any additional labeling or annotation of images at the target site. In this paper, our team investigates the use of two approaches for performing the adaptation: (1) color normalization and (2) adversarial training. The adversarial training strategy is implemented through the use of convolutional neural networks to find an invariant feature space and Siamese architecture within the target domain to add a regularization that is appropriate for the entire set of whole-slide images. The adversarial adaptation results in significant classification improvement compared with the baseline models under a wide range of experimental settings.

## 1. Introduction

Advances in whole-slide scanner technology have increased the speed and reliability with which histopathology slides and other microscopic specimens are digitized. As a result of these improvements, there has been a sharp increase in the number of investigators and health-care providers adopting the use of these devices in routine research and clinical workflows. The sheer volume of digitized specimens now being generated at both small and large institutions has grown accordingly. Once digitized, these specimens are well suited for the application of sophisticated pattern recognition and machine-learning algorithms and strategies that can facilitate automated decision-support and computer-assisted diagnosis. Over the course of hundreds of years, scientists and pathologists have gone to great length to develop and optimize staining methods that augment and enhance the contrast of biological components of interest within these samples at the tissue, cell and sub-cellular levels. Hematoxylin & Eosin (H&E) is a popular stain that is applied to specimens, routinely, that results in nuclei exhibiting a bluish color with cytoplasmic regions rendered in pink (Titford and Bowman, 2012). In spite of the best efforts of the technicians preparing the specimens, however, slight variations in the manner in which these stains are applied to specimens often results in histopathology sections that are inconsistent in visual appearance and samples often containing processing artifacts. While there have been many attempts to completely standardize these methods, the current technology still grapples with these challenges (Reinhard et al., 2001; Gurcan et al., 2009; Khan et al., 2014; Li and Plataniotis, 2015). Since these inherent issues described can lead to variations in the results obtained using image-based quantification approaches to analyze the specimens, our team has been investigating new methods to remove color variation across digitized specimens originating from different institutions as well as batches of imaged specimens that may have been acquired at a single institution at different time points. In earlier attempts to mitigate the color normalization issue, some investigators chose to convert the color images into gray-scale versions before performing quantitative analysis (Hamilton et al., 1997; Jafari-Khouzani and Soltanian-Zadeh, 2003; Ruiz et al., 2007; Qureshi et al., 2008; Basavanhally et al., 2010). However, the conversion from color space to grayscale eliminates some informational content from the digitized specimens that may be essential for rendering proper classifications and accurate diagnosis.

While the noted color variations in digital specimens present formidable technical challenges for any image analysis algorithm, mechanical distortions that can sometimes be introduced during tissue sectioning and slight variations in the underlying morphologic and structural patterns within imaged specimens can further complicate the process of automating classifications (Jafari-Khouzani and Soltanian-Zadeh, 2003; Tabesh et al., 2007; Ren et al., 2015, 2017; Epstein et al., 2016; Lafarge et al., 2017). In spite of all of the difficulties, investigators throughout the scientific community continue to pursue this line of research because of the potential impact that automated, computer-aided analyses could have in clinical practice and investigative research by accelerating the throughput while reducing or eliminating the negative effect of inter- and intra-observer variations during the assessment of microscopic images. Methods based on convolutional neural networks (CNN) are currently considered state-of-the-art due to the high performance rates recently reported by some recent investigations (Otálora et al., 2015; Hou et al., 2016; Litjens et al., 2016). Most of these studies, however, focused on supervised classification. Unfortunately, supervised classification models used on one annotated dataset (source domain) may render ineffective for another set (target domain) collected at a different institute. A widely used approach to address the challenge is to label new images on the target domain and fine-tune the model trained on source domain (Schmidhuber, 2015). In fact, methods that can learn from existing datasets and adapt to new target domains, without the need for additional labeling, are among the most desirable approaches because they lend themselves to high-throughput clinical environments and big data research experiments involving large patient cohorts (Ren et al., 2018).

In this study, we aim to address the challenges presented by variations in staining, morphologic and architectural profiles within histopathology whole-slide images (WSIs) in a completely unsupervised manner. We use two approaches to achieve knowledge transfer from the source domain to the target domain. In the first approach, we adopt two off-the-shelf color normalization (Macenko et al., 2009; Vahadane et al., 2016) on the images from the target domain, where the model learned from the source domain is applied to the target images after being normalized to the reference image chosen from the source domain. In the second approach, we adopt an unsupervised domain adaptation paradigm to align the image distributions along the annotated source domain and the unlabeled target domain (Ganin et al., 2016; Tzeng et al., 2017). We apply adversarial training to minimize the distribution discrepancy in the feature space between the domains, using the loss function adopted from the Generative Adversarial Network (Goodfellow et al., 2014). We subsequently develop a Siamese architecture for the target network to serve as a regularization of patches within the WSI's. We validate the proposed methods on a set of publicly available histopathology datasets and then further test performance using a new dataset that is collected locally at Rutgers Cancer Institute of New Jersey. The experimental results show the merit of these strategies.

## 2. Related Works

### 2.1. Color Normalization

In an attempt to address the challenge of the previously described color batch effects, many investigators have applied color normalization methods to the imaged histopathology specimens prior to analysis (Ranefall et al., 1997; Meurie et al., 2003; Mao et al., 2006; Kong et al., 2007; Kothari et al., 2011; Khan et al., 2014; Tam et al., 2016; Vahadane et al., 2016; Alsubaie et al., 2017; del Toro et al., 2017; Janowczyk et al., 2017; Gadermayr et al., 2018; Sankaranarayanan et al., 2018; Zanjani et al., 2018a). One common approach for analyzing tissue samples is to treat stains as agents exhibiting selective affinities for specific biological substances. With an implicit assumption that the proportion of pixels associated with each stain is same in source and target images, histogram-based methods are investigated (Jain, 1989; Kong et al., 2007; Tabesh et al., 2007; Hipp et al., 2011; Kothari et al., 2011; Papadakis et al., 2011; Krishnan et al., 2012; Basavanhally and Madabhushi, 2013; Bejnordi et al., 2016; Tam et al., 2016). The main drawback of histogram-based methods is that they often introduce visual artifacts into the resulting images. Color deconvolution strategies (Macenko et al., 2009; Niethammer et al., 2010; Gavrilovic et al., 2013) have been utilized extensively in the analysis imaged histopathology specimens by separating RGB images into individual channels such as by converting from RBG to Lab (Reinhard et al., 2001) or HSV space (Zarella et al., 2017). The limitation of this approach is that both the image-specific stain matrix and a control tissue stained with a single stain is required to perform the color deconvolution. Another strategy that has been explored is to utilize blind color decomposition which is achieved by applying expectation and maximization operations on color distributions within the Maxwell color triangle (Gavrilovic et al., 2013). This strategy requires a heuristic randomization function to select stable colors for performing the estimation, thus it is prone to be affected by achromatic pixels at the weak stain pixels. Tissue inherent morphological and structural features may not be preserved after color deconvolution since statistical characteristics of decomposition channels are modified during this process. Model-based color normalization has also been studied in such applications by including Gaussian mixture models (Reinhard et al., 2001; Magee et al., 2009; Basavanhally and Madabhushi, 2013; Khan et al., 2014; Li and Plataniotis, 2015), matrix factorization (Vahadane et al., 2016), sparse encoder (Janowczyk et al., 2017), and wavelet transformation with independent component analysis (Alsubaie et al., 2017). Other studies utilize generative models (Goodfellow et al., 2014) to achieve the stain normalization (Cho et al., 2017; Bentaieb and Hamarneh, 2018; Shaban et al., 2018; Zanjani et al., 2018b). Typically, a reference image is needed from a group of image dataset. The different reference image would give the different domain adaptation performance. Color normalization models can provide stain estimation, but they are solely dependent on image color information, while the morphology and spatial structural dependency among imaged tissues is not considered (Gavrilovic et al., 2013; Bejnordi et al., 2016; Tam et al., 2016; Zarella et al., 2017), which could lead to unpredictable results especially when strong staining variations appear in the imaged specimens.

### 2.2. Adversarial Domain Adaptation

In recent years, there have been many studies on unsupervised domain adaptation for transferring the learned representative features from the source to the target domain (Bousmalis et al., 2017; Herath et al., 2017; Wu et al., 2017; Yan et al., 2017). The works based on CNN show significant advantages due to better generalization across different distributions (Krizhevsky et al., 2012; Luo et al., 2017). With the development of the Generative Adversarial Networks (GAN) (Goodfellow et al., 2014), studies show the synthesized images could be used to perform unsupervised domain adaptation in a learned feature space where a generator is applied to learn the image distribution and generate the synthetic images while a discriminator is trained to differentiate the synthesized and the real distribution (Bousmalis et al., 2016; Liu and Tuzel, 2016). For example, Generate-to-Adapt (Sankaranarayanan et al., 2018) proposes to learn a joint embedding space between the source and target domain, where the embedding space could be used to synthesize both the source and target images. Inspired by previous studies, we utilize the adversarial training to find a discriminative feature space that can be used to transfer the knowledge from source to target domain. Furthermore, we introduce a Siamese architecture at target domain which can be used to regularize the classification of WSIs in an unsupervised manner.

## 3. Materials

**Figure 1:**
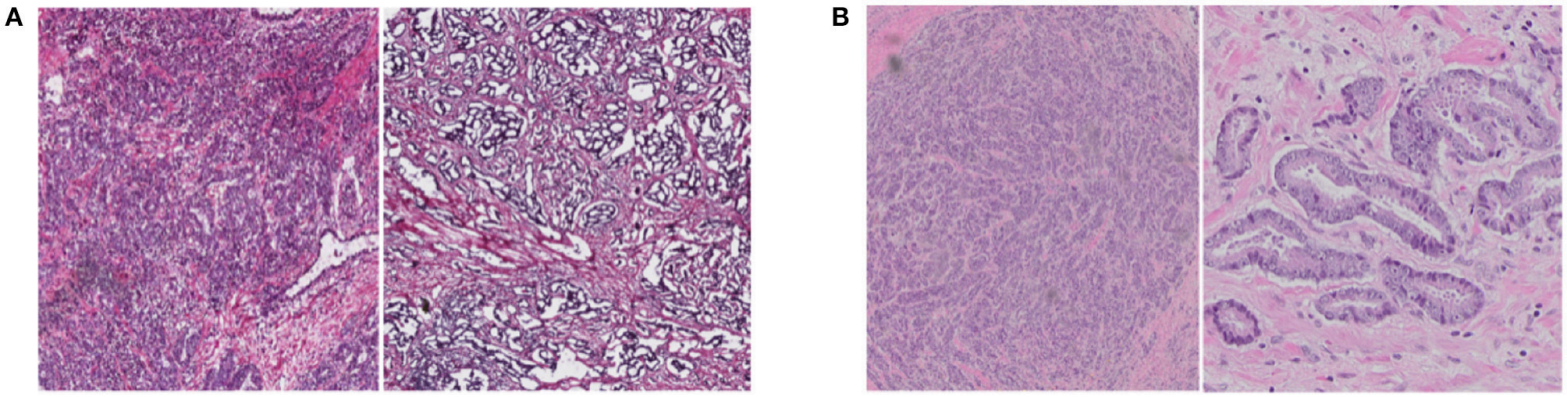
Examples of prostate cancer histopathology WSIs from TCGA **(A)** and RCINJ **(B)**. The WSIs from different institutes present different glandular distribution and staining appearance.

For the purposes of the current study, we focus on unsupervised domain adaptation of imaged prostate cancer histopathology specimens. Prostate cancer is the most common non-cutaneous malignancy afflicting 1 in 7 men in the United States (Ferlay et al., 2015). Over the years, Gleason scores have consistently served as a reliable predictor for differential prostate cancer diagnosis (Epstein et al., 2016). Unfortunately, Gleason grading can be extremely time-consuming when attempting to systematically evaluate large, giga-pixel-sized WSIs. Furthermore, inter- and intra-observer variability errors often arise when pathologists are called upon to render diagnoses based on WSIs. In order to provide an objective and reproducible Gleason grading score on such datasets, reliable computational methods are required for detection, extraction, and recognition of the underlying histopathological patterns. Much of the progress in this area of research has focused on supervised classification of the imaged tissues (Doyle et al., 2007; Tabesh et al., 2007; Khurd et al., 2011; Nguyen et al., 2012; Gorelick et al., 2013). However, the fact that histopathology WSIs obtained from different institutions often present divergent glandular appearances due to the fact that the acquisitional and optical properties of the specific type of scanners used and differences in the sectioning and staining procedures utilized introduce significant variations in the resulting images. Additionally, WSIs scanned by from different institution may have different image resolution as they were scanned under various microscopy. Figure 1 shows representative prostate cancer tissue images originating from different institutions. Note the variations in glandular distributions and staining appearance.

Our team investigated the use of unsupervised domain adaptation for histopathology images and tested the approach on two datasets. The first which is publicly available is called The Cancer Genome Atlas (TCGA) dataset (Kandoth et al., 2013). The other is a dataset collected locally at Rutgers Cancer Institute of New Jersey (RCINJ) after obtaining institutional review board (IRB) approval. All the histopathology images are H&E stained. For the first setting of unsupervised domain adaptation, we only use the TCGA dataset. The TCGA prostate cancer dataset includes histopathology WSIs uploaded from 32 institutions that have been acquired at 40 × and 20 × magnifications. We crop the WSIs into patches of size 2,048 × 2,048. We calculate the tissue area on the grayscale images and remove the images with tissue area less than half of the patch size. The dataset includes Gleason scores, ranging from 6 to 10, that have been annotated by pathologists. As the University of Pittsburgh (UP) had contributed more images than other institutions, we treat the UP images as the target domain where the annotations are withheld and the images from other institutions as the source domain, which we denote as TCGA (w/o UP). We show the total number of WSIs and the cropped patches from TCGA in Table 1 and UP in parentheses. We denote the adaptation setting as TCGA (w/o UP) → UP. For the second setting of the unsupervised domain adaptation, we use all the images from TCGA as the source domain, and images from RCINJ as the target domain. The images from RCINJ are acquired at 20 × magnification. More details of the RCINJ dataset are shown in Table 2. The dataset was labeled as Gleason scores as 6 or 8 by a board-certified pathologist. We denote this adaptation as TCGA → RCINJ.

**Table 1:** The number of WSIs and patches of the prostate histopathology images from TCGA under different Gleason scores.

	**Gleason 6**	**Gleason 7**	**Gleason 8**	**Gleason 9**	**Gleason 10**
# WSIs	115 (32)	395 (95)	94 (20)	128 (24)	4 (0)
# Patches	16,293 (6,517)	67,162 (26,583)	16,204 (4,968)	23,978 (9,606)	342 (0)

*The images from University of Pittsburgh (UP) are shown in parentheses*.

**Table 2:** The number of WSIs and patches of the prostate histopathology images from RCINJ under different Gleason scores.

	**Gleason 6**	**Gleason 8**
# WSIs	57	26
# Patches	3,933	666

For the two sets of unsupervised adaptation, we aimed to transfer the knowledge gained from the source image data to the images in target domain so that a network could reliably classify the WSIs in the target domain into low- and high-Gleason score categories. Specifically, the methods were used to divide the TCGA dataset into low Gleason grade for the WSIs with score as 6 and 7, and high Gleason grade for the WSIs with score as 8, 9, and 10. In the case of the RCINJ dataset, the WSIs with Gleason score of 6 belong to the low-Gleason grade whereas those assigned a Gleason score of 8 belonging to high Gleason grade.

## 4. Methods

In this section, we introduce the two different unsupervised methods to solve the domain variation necessary for rendering accurate classification of histopathology images.

### 4.1. Problem Formulation

For the purposes of the experimental design, the annotated images are established at source domain whereas the unlabeled images are housed at the target domain. To facilitate the study, for the source domain, we denote S as the image distribution, *N*_*s*_ as the total number of annotated images, ${\left\{\left({\text{x}}_{i}^{s},{\text{y}}_{i}^{s}\right)\right\}}_{i=1}^{N_{s}}$ as the *i*^*th*^ image **x**^*s*^ with the one-hot category information of **y**^*s*^. Similarly, for the target domain, we denote T as the image distribution, *N*_*t*_ as the total number of unlabeled images, ${\left\{\left({\text{x}}_{i}^{t}\right)\right\}}_{i=1}^{N_{t}}$ as the *i*^*th*^ image unlabeled image **x**^*t*^.

We use the images from the source domain to learn a mapping function *M*_*s*_ that can reliably transform the images to the feature space. Then we apply two approaches for the unsupervised domain adaptation. The first transfers the staining information from the images of the source domain to the images of the target domain so that the classification of target domain can be easily achieved by using *M*_*s*_. The second identifies the mapping *M*_*t*_ that must occur at the target domain to obtain a similar feature space to that found within the source domain. The prediction for images at the target domain can be obtained by using *M*_*t*_ directly. Each domain makes use of training, validation and test sets while the labels for the training images in the target domain are withheld.

### 4.2. Learning at Source Domain

Images from the source domain are annotated and the classification of each is independently confirmed by a board-certified pathologist. These images are subsequently used to teach the source domain CNN to map the images into a discriminative feature space. Due to the giga-pixel size of histopathology WSI, each was cropped into manageable sized patches and the cross-entropy loss was adopted $L_{\text{c}}$ to optimize the performance of the classifier **C** in a supervised manner.

(1) $$\begin{array}{r} L_{\text{c}}=E_{{\text{x}}^{s}\sim S}-\overset{N_{s}}{\underset{i=1}{\sum }}{\text{y}}_{i}^{s}\cdot \text{log}\text{C}\left(M_{s}\left({\text{x}}^{s};\theta^{S}\right)\right). \end{array}$$

**Figure 2:**
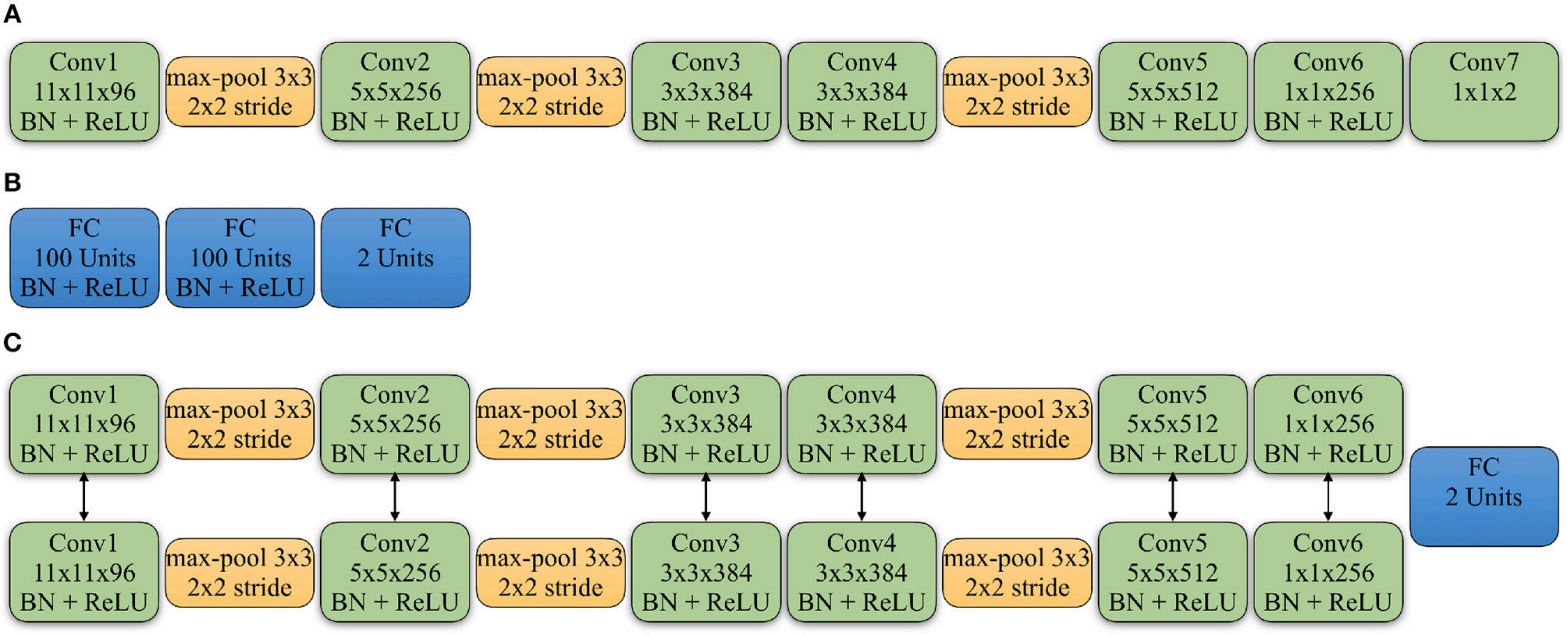
Detailed architectures of source domain network, discriminator and Siamese network of target network: **(A)** The convolutional neural network applied in the source domain. All the convolution layers (Conv) are followed by the Batch Normalization layer (BN) and Rectified Linear Units (ReLU), except for the last Conv layer that gives the classification. The Conv5 and Conv6 layers are also followed by a Dropout layer with the ratio as 0.5. **(B)** The architecture of the discriminator. All the FC layers are followed by the BN and ReLU, except for the last FC layer that gives the domain prediction. **(C)** The Siamese network applied in the target domain. The Conv5 and Conv6 layers from the two branches are followed by a Dropout layer with the ratio as 0.5. And the two branches share the same parameters. The feature maps from Conv6 are concatenated to feed into a FC layer to give the similarity prediction between input patches. The Conv6 layers are also followed by a Conv7 layer with the same kernel size as shown in the source domain CNN.

In the above equation, θ^*S*^ represents the weights of the source domain CNN. We used a modified fully convolutional AlexNet (Krizhevsky et al., 2012) as the source domain CNN for the classification task. The network does not include a fully connected (FC) layer, instead it only contains convolutional layers. All of the convolutional layers are followed by the Batch Normalization layer (Ioffe and Szegedy, 2015) and Rectified Linear Units (ReLU), except for the last layer that provides the actual prediction. The details of the network are shown in Figure 2A. To achieve the classification for the WSIs, we apply a majority vote on all cropped patches within each WSI which, in turn, provides the prediction.

Due to the high number of domain variations that are exhibited in histopathology images, the network learned from the source domain may not always generalize sufficiently within the target domain. To address this issue, we introduced two approaches to minimize the domain variations with the details followed.

### 4.3. Color Normalization for Target Domain

The first approach for achieving unsupervised domain adaptation in the histopathology images of target domain utilizes the color normalization. As it can be applied to improve the automated diagnostic performance of histopathology images by decreasing the staining variation among the entire cohort (Ren et al., 2015; Ciompi et al., 2017; Bentaieb and Hamarneh, 2018; Zanjani et al., 2018b).

In order to apply the source mapping *M*_*s*_ on the target domain directly, we transfer the H&E staining information from source domain to the target domain by normalizing the target images according to a reference image chosen from the source domain. In this case, only the test images from target domain are required to validate the performance while the training images from the target domain are withheld. However, choosing the reference image from source domain is a non-trivial process given the large number of candidate images. Therefore, we uniformly sample a total of *N*_*l*_ reference images from source domain. For each image **x**^*t*^ in the target domain, we normalize it using each reference image and forward the normalized image ${\text{x}}_{j}^{t}$ into the source domain CNN to generate the logits feature vector. Then we adopt unweighted averaging, as it has been shown as a reasonable ensemble method in deep learning networks (Simonyan and Zisserman, 2014; He et al., 2016), to construct the ensemble logits feature **l**_*N*_*l*__ of **x**^*t*^ for the *N*_*l*_ iterations, as shown below:

(2) $$\begin{array}{r} {\text{l}}_{N_{l}}=\frac{1}{N_{l}}\overset{N_{l}}{\underset{j=1}{\sum }}M_{s}\left({\text{x}}_{j}^{t};\theta^{S}\right). \end{array}$$

Thus the class prediction for **x**^*t*^ could be achieved by using *softmax* on **l**_*N*_*l*__. In this study, we apply two color normalization methods, which are Macenko (Macenko et al., 2009) and SPCN (Vahadane et al., 2016), as their advantages have been shown in histopathology images (Roy et al., 2018).

### 4.4. Adversarial Adaptation for Target Domain

The color normalization process makes it possible to perform the stain transfer from source domain to target domain on images directly. The second approach we investigated was unsupervised domain adaptation of histopathology images, in which we explored the adaptation of knowledge on feature space from source to target domain. Therefore, we learn a target mapping function *M*_*t*_, which is a CNN, to map the images from target domain into a discriminate feature space. In order to optimize the target network, we leverage the adversarial training to minimize the discrepancy between the feature space of the target domain and the one of the source domain. We perform asymmetric adaptation where the network at the target domain is fine-tuned from the network of the source domain. Through optimization, the feature space of the target domain learns to mimic the distribution of the source feature space. Thus, the target network is trained to extract the domain invariant features from input samples, which have the same distribution as the source domain. In the process, the training images of target domain are used to carry out the adversarial adaptation.

#### 4.4.1. Adversarial Training

We implement adversarial training following the idea from GAN loss (Goodfellow et al., 2014) on the feature spaces of source and target domain. The feature vectors generated from the network of source domain or the network of target domain are fed into the discriminator **D**. **D** is trained to map the input feature vectors into a binary domain label, where the “true” denotes the input feature vectors are from source domain and “false” denotes the feature vectors are from target domain. Additionally, the target mapping *M*_*t*_ is learned in an adversarial manner to purposely misdirect the discriminator **D** by reversing the domain label so that the discriminator cannot distinguish between the two feature spaces. Since the mapping parameterization of source model is determined before the adversarial training, we only optimize the target mapping step *M*_*t*_. By using adversarial learning, we minimize the discrepancy of feature spaces between the source and target domain. Therefore, estimating the category information for the images from target domain can be implemented by *M*_*t*_. More specifically, the adversarial loss $L_{{\text{adv}}_{\text{D}}}$ for optimizing the discriminator **D** is represented as:

$$\begin{array}{rcl} \underset{\text{D}}{\text{min}}L_{{\text{adv}}_{\text{D}}} & = & -E_{{\text{x}}^{s}\sim S}\text{log}\text{D}\left(M_{s}\left({\text{x}}^{s};\theta^{S}\right);\theta^{D}\right) \end{array}$$

(3) $$\begin{array}{r} \underset{D}{\text{min}}{ℒ}_{{\text{adv}}_{D}}=-E_{x^{s}\sim S}\text{log}D(M_{s}(x^{s};\theta^{S});\theta^{D})\\ \;-E_{x^{t}\sim T}\text{log}(1-D(M_{t}(x^{t};\theta^{T});\theta^{D}). \end{array}$$

where θ^*T*^ represents the weights of the target domain CNN and θ^*D*^ represents the weights of the discriminator. The discriminator is composed of three fully connected layers where each is followed by a Batch Normalization layer and a ReLU layer with the exception of the last one. The details for the architecture of the discriminator are shown in Figure 2B. The mapping loss $L_{{\text{adv}}_{M}}$ for optimizing the target mapping *M*_*t*_ is represented as:

(4) $$\begin{array}{r} \underset{M_{t}}{\text{min}}L_{{\text{adv}}_{M}}=-E_{{\text{x}}_{t}\sim T}\text{log}\left(\text{D}\left(M_{t}\left({\text{x}}^{t};\theta^{T}\right);\theta^{D}\right)\right). \end{array}$$

For the adversarial training, we optimize the $L_{\text{a}}$, where $L_{\text{a}}=L_{{\text{adv}}_{\text{D}}}+L_{{\text{adv}}_{M}}$.

#### 4.4.2. Siamese Architecture for Target Network

**Figure 3:**
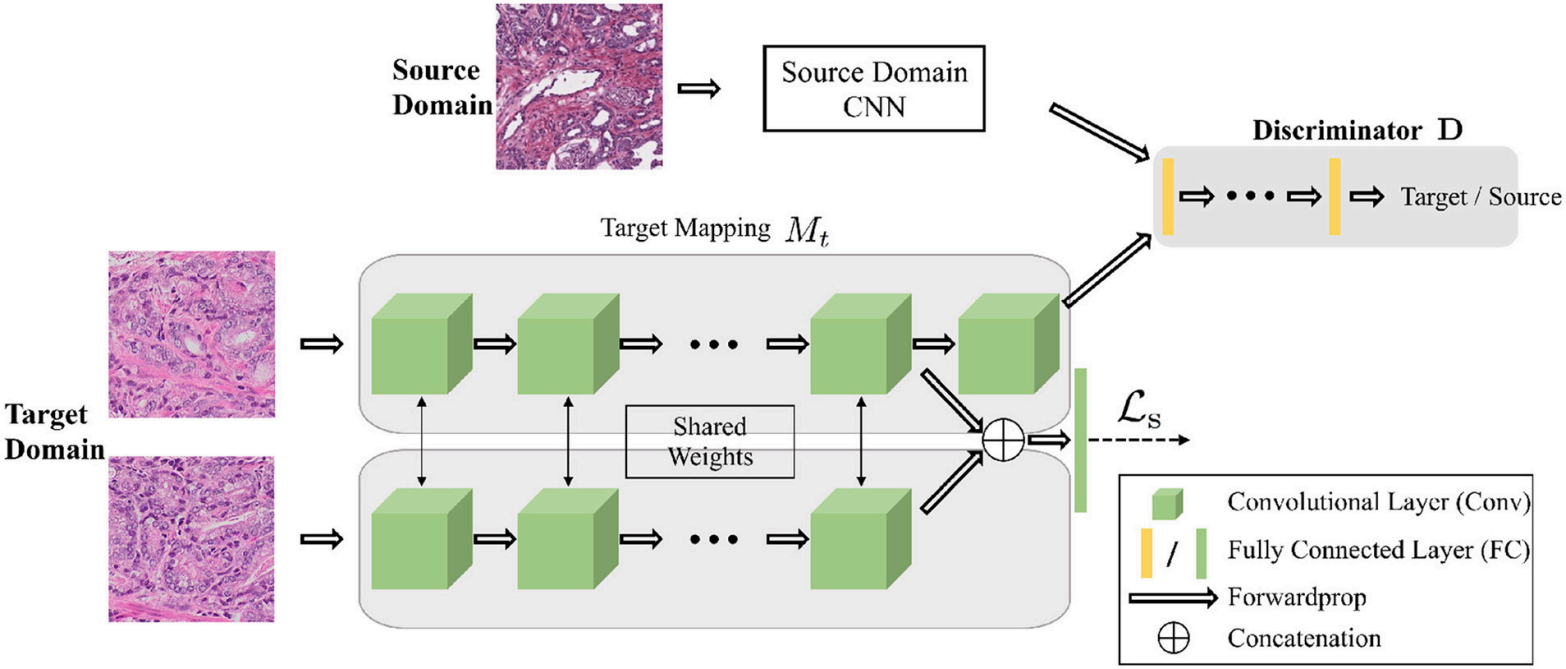
The architecture of the networks for the adversarial domain adaptation. The source network and the target network map the input samples into the feature space. The adaptation is accomplished by jointly training the discriminator and target network using the GAN loss to find the domain invariant feature. A Siamese network at target domain adds constrains for patches within the same WSIs.

Although there are no annotations for the images at the target domain, the patches cropped from the same WSI should be estimated as the same class by the network at target domain. However, the adversarial loss only forces the distribution of the feature spaces across the two domains to be similar, it can not constrain the target network to determine the similarity of the input samples. Therefore, we introduce a Siamese architecture (Chopra et al., 2005) at target domain to explicitly regularize patches from the same WSI to be classified into the same category. As shown in Figure 3, the two identical networks in the target domain share the same weights with the input as a pair of images (${\text{x}}_{1}^{t}$, ${\text{x}}_{2}^{t}$) ⊆T×T. The feature maps obtained from the second to the last layer of the two networks, namely the Conv6 feature maps as shown in Figure 2C, are concatenated together to serve as the input vector for a one-layer perceptron to classify the features. Therefore, the input samples are classified by the function $f\left({\text{x}}_{1}^{t},{\text{x}}_{2}^{t};\theta^{F}\right)$, that $f:T\times T\mapsto \bar{\text{y}}$ and θ^*F*^⊆θ^*T*^, where $\bar{\text{y}}$=1 indicates input patches belong to the same WSI while $\bar{\text{y}}$=0 denotes not. We learn the binary classifier *f* using categorical cross-entropy loss $L_{\text{s}}$ as following:

(5) $$\begin{array}{r} L_{\text{s}}=E_{\left({\text{x}}_{1}^{t},{\text{x}}_{2}^{t}\right)\sim T}-\overset{N_{p}}{\underset{i=1}{\sum }}{\bar{\text{y}}}_{i}\cdot f\left({\text{x}}_{i1}^{t},{\text{x}}_{i2}^{t};\theta^{F}\right). \end{array}$$

where *N*_*p*_ denotes the total number of training pairs.

To learn the network at target domain by adversarial adaptation, we adopt a two-stage training process. For the first stage, we train the network at source domain, which is the same as using the color normalization in the adaptation process. For the second stage, we optimize the Siamese network at target domain by applying $L_{\text{t}}$ where $L_{\text{t}}=L_{\text{a}}+L_{\text{s}}$. For optimizing $L_{\text{s}}$, we sample the images pairs in the training set of target domain both from the patches cropped from the same WSI and the patches from different WSIs. The learning algorithm for the target network is shown in Algorithm 1.

**Algorithm 1:** Learning Algorithm for the Network at Target Domain

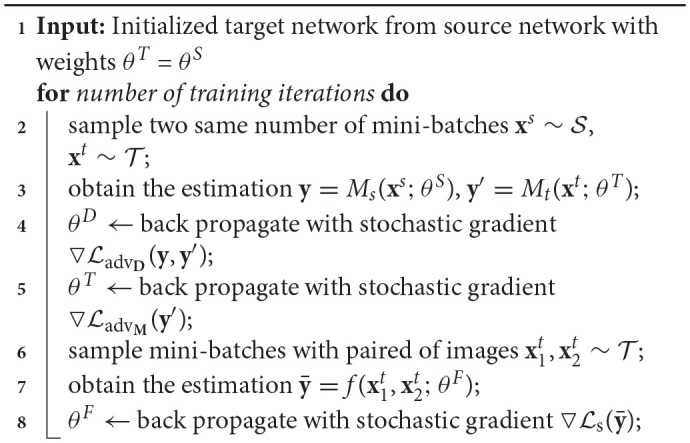

## 5. Experiments

In this section, we validate the proposed approaches using the unsupervised domain adaptation for the classification of the histopathology images.

### 5.1. Implementation Details

We conducted two sets of unsupervised domain adaptation for classification of prostate histpathology images, which are TCGA (w/o UP) → UP and TCGA → RCINJ. We firstly use the images in source domain to train a binary classification network. The data from source domain is randomly divided into the training and the testing sets at a ratio of 80% (validation set is randomly selected from the training set) / 20%. The patients with more than one WSI can only contribute the images to the training set or the testing set. During the training process, the images are resized as 256 × 256 and randomly cropped to 224 × 224 to feed into the network. During the testing process, all the patches are resized to 256 × 256, we do the single center-crop for all testing patches. The network is trained from scratch. For the adaptation using color normalization, we utilize the source domain CNN as the network for target domain to determine the prediction from the testing set. For the adversarial adaptation, we optimize the Siamese network at target domain by fixing the parameters of source domain CNN and training the target network and the discriminator network at the same time. The prostate images at the target domain are randomly divided into the training and the testing sets at a ratio of 80 and 20%.

Our implementation is based on Tensorflow (Abadi et al., 2016). To train the source network, we use mini-batch Stochastic Gradient Descent (SGD) with mini-batch size as 128. The momentum is 0.9 and the weight decay is 0.0005. The initial learning rate is 0.001 and periodically annealed by 0.1. To train the target network for the adversarial adaptation, we use Adam optimization (Kingma and Ba, 2014) with the fixed learning rate as 0.00001. The mini-batch size for optimizing $L_{\text{a}}$ and $L_{\text{s}}$ is set as 128.

### 5.2. Source Domain Performance

As the training process contains two steps, we first show the performance of the network at the source domain. The comparison between the source network and the previous study (del Toro et al., 2017) is shown in Table 3. From the results, we can see both of our models have better performance than (del Toro et al., 2017). However, the study at del Toro et al. (2017) uses less WSIs than ours and the network with the best performance reported in del Toro et al. (2017) is wider and deeper than our study. Although such differences lead to biased comparison, it could still demonstrate the source domain network is well trained to classify the TCGA prostate images into low Gleason score and high Gleason score. We have tried deeper network, such as ResNet-50 (He et al., 2016), but the modified AlexNet used in the study has a better performance. For example, the modified AlexNet has the accuracy of 83.0% on TCGA while the ResNet-50 (He et al., 2016) has the accuracy as 79.8%.

**Table 3:** The source domain network performance.

	**Accuracy (%)**
Previous study (del Toro et al., 2017)	73.5
TCGA (w/o UP)	76.9
TCGA	83.0

*The source domain classification network outperforms previous study (del Toro et al., 2017) using prostate cancer data from TCGA without UP and TCGA. The source domain network using one all TCGA prostate cancer data achieves higher classification accuracy than using TCGA without UP because of more data included for training the network*.

### 5.3. Comparison Results

In this section, we show the comparative results using different approaches for learning the classification model at the target domain.

#### 5.3.1. Adaptation Using Color Normalization

**Figure 4:**
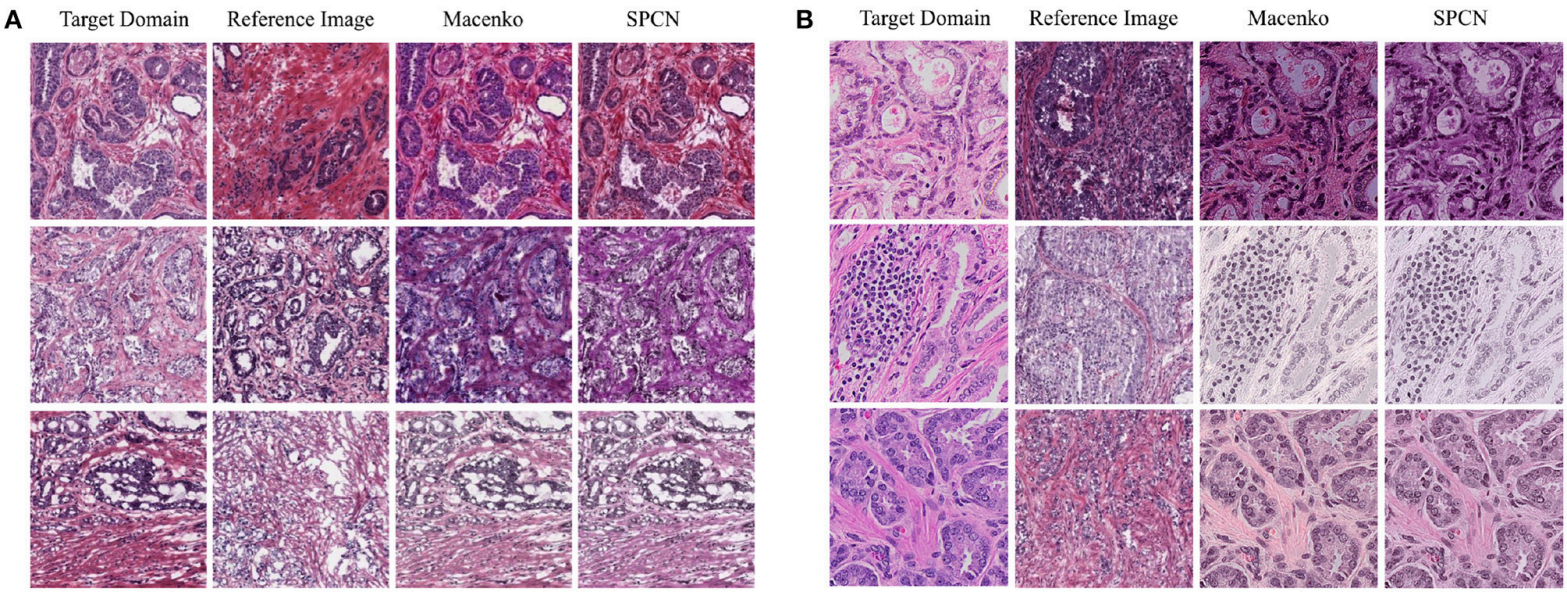
Example images selected from the testing set of target domain are normalized by the reference images sampled from the training set of source domain using two color normalization methods including Macenko (Macenko et al., 2009) and SPCN (Vahadane et al., 2016). **(A)** The adaptation of TCGA (w/o UP) → UP. **(B)** The adaption of TCGA → RCINJ.

First, we show the domain adaptation results only using color normalization. The qualitative results for the color normalization are shown in Figure 4. We sample different number of reference images, which is *N*_*l*_ in Equation 2, due to the large number of training set in source domain. For each color normalization method, we use *N*_*l*_-Ensemble to indicate the number of reference images. For each *N*_*l*_, we run the experiments for 10 times and report the mean and the standard deviation values in Table 4. Additionally, we show the baseline results in Table 4 where the source domain CNN is applied on the original images from target directly. We can see that due to the different image distributions of the source and target domains, the network learned from source domain is not working appropriately when applied on target domain directly. For the adaptation of TCGA (w/o UP) → UP, the results show using the two color normalization methods both improve the classification accuracy and with more reference images, it could achieve the better classification. Furthermore, SPCN (Vahadane et al., 2016) achieves better results compared to Macenko (Macenko et al., 2009) as it has higher mean classification accuracy and less standard deviation. While for the adaptation of TCGA → RCINJ, no better result is observed by using the color normalization, which indicates color normalization may not be robust when applied for the domain adaptation of the prostate histopathology images. For both TCGA (w/o UP) → UP and TCGA → RCINJ, using more reference images could decrease the standard deviation of the ensemble results. On the other hand, the high standard deviation indicates the high sensitivity when choosing a reference image, which makes the color normalization less practicable for unsupervised domain adaptation given the difficulty of deciding the optimal reference image within the source domain.

**Table 4:** Unsupervised domain adaptation for TCGA (w/o UP) → UP and TCGA → RCINJ using color normalization and adversarial adaptation.

	**TCGA (w/o UP) → UP**	**TCGA → RCINJ**
Baseline	54.3	56.3
Macenko (Macenko et al., 2009) 1-Ensemble	65.7 ± 11.9	51.3 ±6.1
Macenko (Macenko et al., 2009) 2-Ensemble	70.0 ± 5.9	53.8 ±8.5
Macenko (Macenko et al., 2009) 5-Ensemble	72.3 ± 3.8	55.0 ± 7.3
Macenko (Macenko et al., 2009) 10-Ensemble	72.6 ± 2.3	55.0 ± 4.7
SPCN (Vahadane et al., 2016) 1-Ensemble	70.0 ± 7.3	56.3 ± 13.4
SPCN (Vahadane et al., 2016) 2-Ensemble	71.7 ± 6.7	55.0 ± 15.3
SPCN (Vahadane et al., 2016) 5-Ensemble	72.9 ± 2.6	55.6 ± 9.8
SPCN (Vahadane et al., 2016) 10-Ensemble	73.4 ± 1.8	54.4 ± 8.4
Color augmentation (Liu et al., 2017)	74.5	56.3
Generate-to-Adapt (Sankaranarayanan et al., 2018)	71.7	62.5
$L_{\text{a}}$ only	71.4± 1.1	62.5 ± 2.5
$L_{\text{t}}$	77.1± 1.1	75.0 ± 2.5

*The classification accuracy of two color normalization methods including Macenko (Macenko et al., 2009) and SPCN (Vahadane et al., 2016) with different number of ensembles, and the target network with adversarial loss ($L_{\text{a}}$) only and the target network with adversarial loass and Siamese loss together ($L_{\text{t}}$) are shown for two sets of adaptations. We also compare our approach with color augmentation (Liu et al., 2017). Our proposed approach has a better performance than other state-of-the-art study (Sankaranarayanan et al., 2018) on the unsupervised adaptation task*.

Additionally, we show the comparison with color augmentation, which has been proved effective for the data augmentation of histopathology images (Liu et al., 2017; Nazeri et al., 2018; Rakhlin et al., 2018). We follow the methods introduced in Liu et al. (2017) where random color perturbations is applied on each patch in the training set. Experimental results in Table 4 show the color augmentation is more effective than color normalization on the two sets of experiments.

#### 5.3.2. Adversarial Adaptation

Second, we show the results of using the adversarial domain adaptation for TCGA (w/o UP) → UP and TCGA → RCINJ. The quantitative results for the adaptation are shown in Table 4. Through the adversarial adaptation, we could effectively adopt the discriminative knowledge from TCGA (w/o UP) to the UP and from TCGA to RCINJ without requiring additional annotations. Compared with the adaptation using color normalization, the adversarial adaptation achieves better classification results for the two setting of experiments, which demonstrates its effectiveness and robustness. Additionally, we compare our approach with the Generate-to-Adapt (Sankaranarayanan et al., 2018) on the two tasks and our approach outperforms the current, state-of-the-art algorithm of the unsupervised domain adaptation.

**Figure 5:**
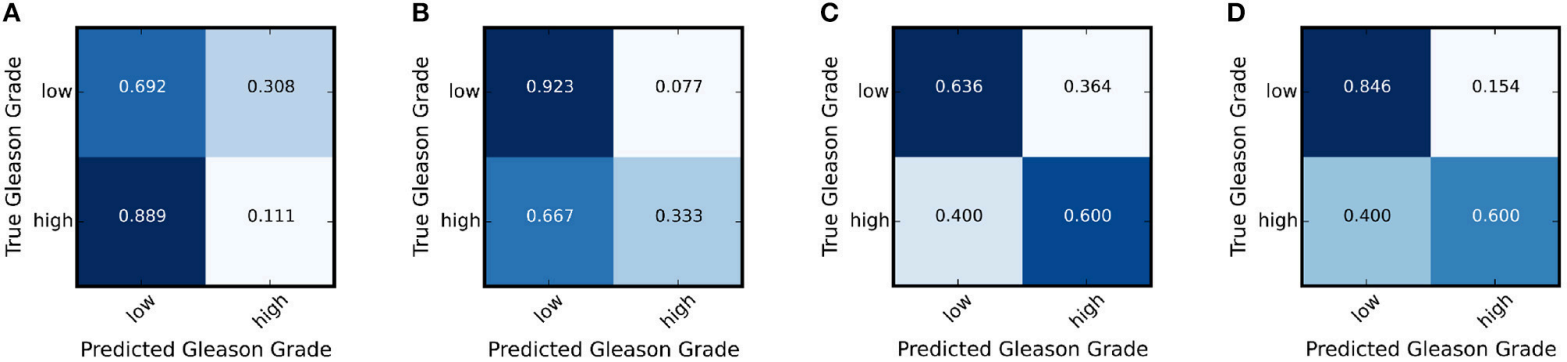
The confusion matrix of the target network before and after the adaptation for TCGA (w/o UP) → UP and TCGA → RCINJ. **(A)** The confusion matrix for UP before domain adaptation. **(B)** The confusion matrix for UP after domain adaptation. **(C)** The confusion matrix for RCINJ before domain adaptation. **(D)** The confusion matrix for RCINJ after domain adaptation.

We further calculate the statistically significance of the accuracy improvement between the adapted network and the baseline network using McNemar Test (Fagerland et al., 2013) and demonstrates the improvement of classification accuracy is statistically significant with a *p* < 0.05. In addition, we show the result of the ablation study in Table 4 that using $L_{\text{t}}$ achieves better classification accuracy than $L_{\text{a}}$ only. Figures 5A,B show the confusion matrices for the adaptation for TCGA (w/o UP) → UP and Figures 5C,D show the confusion matrices for the adaptation of TCGA → RCINJ. Compared to before domain adaptation and after domain adaptation, the true low-grade classification accuracy are significantly improved. It is crucial for prostate cancer diagnosis for patients with low Gleason grade is one of the main criteria for active surveillance and intervention.

**Figure 6:**
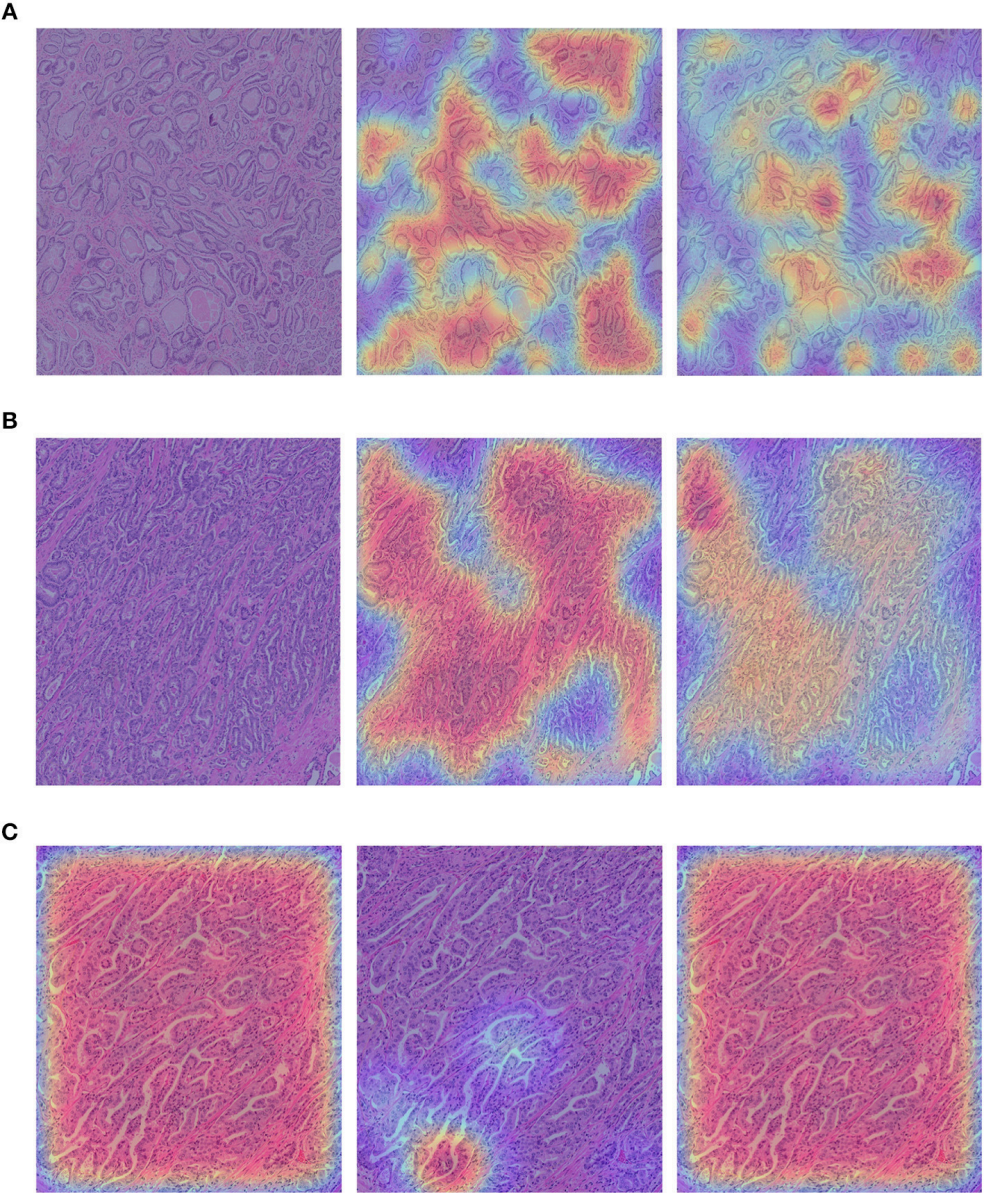
**(A,B)** show the example images from RCINJ with Gleason score 6. **(C)** shows the example image from RCINJ with Gleason score 8. The left column shows the original images with heatmaps overlaid on them; the middle column shows the heatmaps generated from the baseline model (using source domain network); the right column shows the heatmaps generated from the model optimized by $L_{\text{t}}$.

We show the qualitative results for TCGA → RCINJ in Figure 6. We use the probability predicted by the network on the patches to generate a classification probability heatmap and overlay the heatmap on the original image. The red color indicates the high Gleason score and blue color indicates the low Gleason score. Figures 6A,B show example prostate WSIs from RCINJ with the low Gleason score and the ground-truth heatmap overlaid on it. Figure 6C shows the WSI with high Gleason score. After the unsupervised domain adaptation, the target network could correctly classify most of patches into the correct Gleason score.

## 6. Discussion and Conclusion

In this paper, we investigate viable approaches for addressing the challenges presented by the heterogeneous characteristics exhibited within digitized specimens, that arises when analyzing samples that have been prepared at disparate laboratories and institutes. We present two different unsupervised domain adaptation methods to resolve the domain variations to make it possible to render accurate classification of imaged histopathology specimens. To meet the requirements of this endeavor required color normalization to transfer the staining information from images in source domain to the images in target domain whereas adversarial training was implemented to transfer the discriminate information in feature space from the source to the target domain. Throughout these experiments, our team utilized a well-trained CNN at source domain that was shown to outperform other methods used on the TCGA prostate cancer dataset. This work shows that when compared with color normalization, adversarial training is more robust for performing unsupervised domain adaptation, indicating that adversarial training may also serve to decrease the differences in the morphologic and structural patterns for histopathology images that can be introduced during processing at disparate institutions. In this research, we further proposed to leverage a Siamese architecture to add the regularization for the target domain to achieve better results than that resulting from utilizing the state-of-the-art method for unsupervised domain adaptation. Due to the limited size of the datasets in these feasibility studies, we plan to conduct expanded experiments using a wider range of histopathology image classification problems.

## Author Contributions

JR, IH, ES, DF, and XQ conceived the study and wrote the manuscript. JR carried out the experiments.

### Conflict of Interest Statement

ES is the principal investigator on an investigator-initiated clinical trial that is funded by Astellas/Medivation (NCT02885649)^1^. The remaining authors declare that the research was conducted in the absence of any commercial or financial relationships that could be construed as a potential conflict of interest.

## Footnotes

^1^http://cinj.org/clinical-trials/index?show=trial&p=081604
